# Histopathological and parasitological study of the gastrointestinal tract of dogs naturally infected with *Leishmania infantum*

**DOI:** 10.1186/1751-0147-53-67

**Published:** 2011-12-13

**Authors:** Aldair JW Pinto, Maria M Figueiredo, Fabiana L Silva, Trycia Martins, Marilene SM Michalick, Washington L Tafuri, Wagner L Tafuri

**Affiliations:** 1 Departamento de Patologia Geral, Instituto de Ciências Biológicas, Universidade Federal de Minas Gerais, Belo Horizonte, Minas Gerais, Brasil; 2 Departamento de Patologia Veterinária, Escola de Veterinária, Universidade Estadual de Santa Cruz, Santa Cruz, Bahia, Brasil; 3 Departamento de Parasitologia, Instituto de Ciências Biológicas, Universidade Federal de Minas Gerais, Belo Horizonte, Minas Gerais, Brasil; 4 Departamento de Anatomia Patológica e Medicina Legal, Faculdade de Medicina, Universidade Federal de Minas Gerais, Belo Horizonte, Minas Gerais, Brasil

## Abstract

**Background:**

The aim of this study was to provide a systematic pathological and parasitological overview of the gastrointestinal tract (GIT), including the stomach, duodenum, jejunum, ileum, caecum and colon, of dogs naturally infected with *Leishmania*.

**Methods:**

Twenty mongrel dogs naturally infected with *Leishmania (Leishmania) infantum *and obtained from the Control Zoonosis Center of the Municipality of Ribeirão das Neves, Belo Horizonte Metropolitan area, Minas Gerais (MG) state, Brazil, were analyzed. The dogs were divided into two groups: Group 1 comprised nine clinically normal dogs and group 2 comprised 11 clinically affected dogs. After necropsy, one sample was collected from each GIT segment, namely the stomach, duodenum, jejunum, ileum, caecum and colon. Furthermore, paraffin-embedded samples were used for histological and parasitological (immunohistochemistry) evaluation and a morphometrical study were carried out to determine the parasite load (immunolabeled amastigote forms of *Leishmania*). The Friedman and the Mann Whitney tests were used for statistical analysis. The Friedman test was used to analyze each segment of the GIT within each group of dogs and the Mann Whitney test was used to compare the GIT segments between clinically unaffected and affected dogs.

**Results:**

The infected dogs had an increased number of macrophages, plasma cells and lymphocytes, but lesions were generally mild. Parasite distribution in the GIT was evident in all intestinal segments and layers of the intestinal wall (mucosal, muscular and submucosal) irrespective of the clinical status of the dogs. However, the parasite load was statistically higher in the caecum and colon than in other segments of the GIT.

**Conclusion:**

The high parasite burden evident throughout the GIT mucosa with only mild pathological alterations led us to consider whether *Leishmania *gains an advantage from the intestinal immunoregulatory response (immunological tolerance).

## Background

Canine visceral leishmaniasis (CVL) is a worldwide zoonosis prevalent in approximately 50 countries in the Mediterranean basin, Middle East and South America [1]. In Brazil, the parasite *Leishmania infantum *is the cause of CVL [2,3] and the sand fly *Lutzomyia longipalpis *is the principal blood-sucking vector. High infection rates are evident in areas of the country where environmental degradation and disorderly migration of people are related to the urbanization process [4,5]. Dogs are the principal reservoir for the parasite, playing a central role in transmission to humans, and therefore present a serious public health concern [6]. CVL infection is considerably more prevalent than clinical illness in endemic areas; this has been reported both in Brazil and Europe (Mediterranean basin countries) [7-13]. Furthermore, clinical signs of the disease are highly variable and can include lymphadenopathy [14,15], skin lesions [16-18], progressive weight loss [19], hepatosplenomegaly [20,21], ocular and musculoskeletal abnormalities, renal disease and epistaxis [22-24].

Gastrointestinal tract (GIT) disorders occur in human visceral leishmaniasis (HVL) [25] and in dogs both in natural [26-31] and experimental infection [32,33]. Hervás et al. [34] described pathoanatomical alterations in a hemorrhagic stomach during necropsy of a sylvester reservoir in the jackal (*Canis aureus*); diagnosis was confirmed by the presence of macrophages parasitized with amastigotes forms of *Leishmania*. The majority of studies have described clinical signs and the subsequent inflammatory response in canidae predominantly infected with *Leishmania infantum *from the Mediterranean basin. In Brazil, Silva et al. [35] described inflammatory lesions in one clinically unaffected dog that were comparable to those described in the literature. A chronic inflammatory exudate composed of macrophages, plasma cells and lymphocytes, with rare neutrophils and eosinophils, was found throughout the mucosa of the small and large intestine. Immunohistochemical analysis revealed that macrophages of the lamina propria were parasitized with immunolabelled intracellular amastigote forms of *Leishmania*. The authors reported a chronic inflammatory reaction along segments of the GIT involving all histological layers. Subsequently, Toplu and Aydogan [36] carried out a study in 22 dogs naturally infected with *Leishmania *in Turkey and observed a mild to severe mononuclear cell infiltration, predominantly in the lamina propria, in the small and large intestines of all dogs as described by Silva et al. [35]. However, parasites were evident in only 30 percent of cases.

The aim of this study was to provide a systematic pathological and parasitological overview of the GIT of dogs naturally infected with *L. infantum *from an endemic metropolitan area of Belo Horizonte, Minas Gerais, Brazil.

## Methods

### Naturally infected animals

Twenty adult mongrel dogs of unknown age naturally infected with *Leishmania *were identified during an epidemiological survey of CVL carried out by the municipality of Ribeirão das Neves, Belo Horizonte Metropolitan area, Minas Gerais (MG) state, Brazil, using indirect immunofluorescence antibody titers (IFAT) and enzyme-linked immunosorbent assay (ELISA). All dogs were positive for IgG when tested using IFAT (titers > 1:40) and ELISA (Optical Density > 100 > 1:400 dilutions). Clinical examinations were carried out on all the infected dogs, which were subsequently divided into two groups; Group I contained nine dogs that were healthy on clinical inspection (four males and five females), while Group II contained eleven dogs (five males and six females) that exhibited classical signs of CVL, including lymphadenopathy, cutaneous changes (alopecia, dry exfoliative dermatitis or ulcers), onychogryphosis, keratoconjunctivitis, cachexia and anemia.

### Control dogs

Five dogs of unknown age were obtained from the Control Zoonosis Center of the Municipality of Ribeirão das Neves, Belo Horizonte Metropolitan area, Minas Gerais (MG) state, Brazil. Serological (IFAT, ELISA) and parasitological (immunohistochemistry) examinations were negative for *Leishmania *infection.

### Ethical committee approve

In Brazil, animals with canine visceral leishmaniasis are usually not treated. So far, the treatment is prohibited followed by Brazilian Healthy Ministry (Portaria Interministerial 1.426 de 11 de Julho de 2008). According to the Ministry of Health Policy (recommended by World Health Organization) seropositive dogs (ELISA by using Biomanguinhos Test-FIOCRUZ-RJ) are eliminated. Otherwise, we do not euthanize dogs without Ethics Committee approval, particularly control dogs (uninfected dogs). Infected and uninfected dogs were obtained from the Control Zoonosis Center of the Municipality of Ribeirão das Neves, Belo Horizonte Metropolitan area, Minas Gerais state, Brazil. The study was submitted to and approved by the CETEA/UFMG (Comite de Etica em Experimentação Animal/Universidade Federal de Minas Gerais), protocol 218/2009 (valid to March 12, 2013). All procedures involving animals were conducted according to the guidelines of the Colégio Brasileiro de Experimentação Animal (COBEA).

### Necropsy, parasitological diagnosis and histopathology

Dogs were sacrificed with 2.5% (1.0 ml/kg) thiopental (intravenous) and T61™ (0.3 ml/kg). During necropsy, tissue touch preparations (smears) of liver, spleen and lymph nodes were obtained to confirm *Leishmania *infection. Smears were air-dried and stained with Giemsa. Amastigotes forms of *Leishmania *were detected in all animals using oil immersion light microscopy (1000 × magnification). In addition, one sample of each GIT segment, including stomach, duodenum, jejunum, ileum, caecum and colon, were collected, fixed in 10% buffered formalin, dehydrated, cleared, embedded in paraffin, sectioned (3-4 μm thick) and stained with hematoxylin and eosin for histopathological studies. Each GIT sample segment was macroscopically obtained by one fragment. Therefore, each fragment of one TGI sample segment was sliced in other three new samples (transversal sections). All these three transversal sections were paraffin-embedded, cut and disposable in individual histological slide for histological analysis. Blind histological analysis of slides was carried out by a minimum of two pathologists.

### Immunohistochemistry

Immunohistochemistry using the streptoavidin peroxidase reaction was carried out in accordance with Tafuri et al. [37] to demonstrate amastigote forms of *Leishmania *in paraffin-embedded GIT segment tissue samples.

### Morphometrical study

For the histomorphometric study, twenty randomly chosen images (horizontal and vertical movements were carried out using the microscope stage - XY translation in order to avoid overlapping fields) from histological slides of GIT tissue fragments were used to assess the area of immunolabelled amastigotes. A Carl Zeiss image analyzer (KS300 software) as described by Caliari [38] and Lima et al. [39] was utilized, as was an Axiolab light microscope (Zeiss) at a resolution of x440.

### Statistical analysis

The Friedman test was carried out for each segment of GIT within Group I and Group II for statistical analysis.

The Mann Whitney test was performed for comparison of segments between the two groups. The GraphPad Instat 3.0 and Prism 4.0 software's programs were used for these comparisons. In all cases the statistical difference were considered significant when the probabilities of equality p-values were < 0,05.

## Results

Gross examination revealed no severe changes in any part of the GIT mucosa of the dogs included in the study. However, five dogs (20%) (two asymptomatic and three symptomatic dogs) contained helminthes and showed minor focal hyperemia, but no hemorrhages or ulcers were evident.

**Figure 1:**
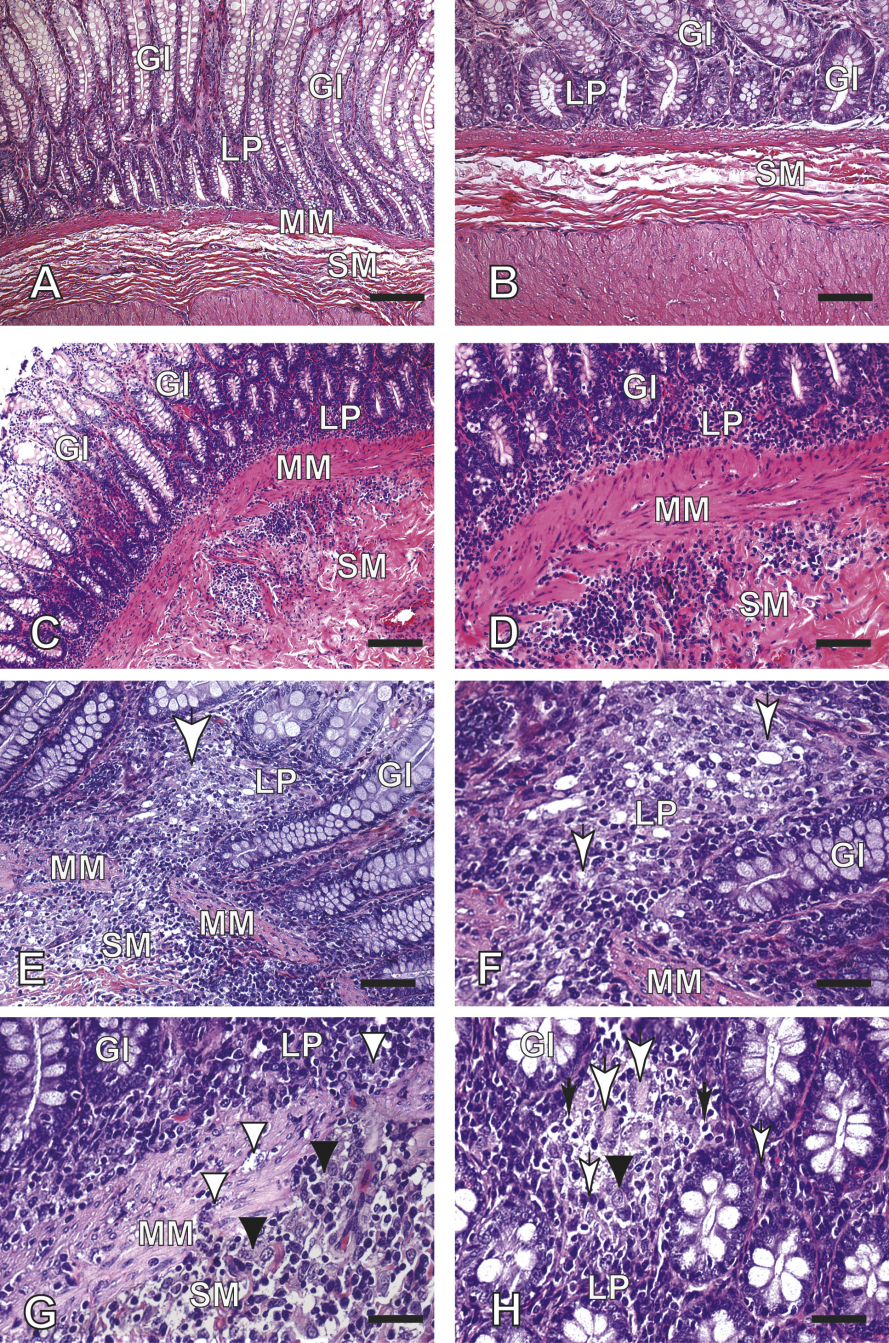
**(A and B) Caecum sections of dogs uninfected (control); (C-H) Caecum sections of dogs naturally infected with *L. infantum *(A and B) Observe a histological normal picture of the mucosal (lamina propria), muscularis mucosae and submucosal layers, HE (Bars = 62 μm and 32 μm, respectively); (C-H) Symptomatic dog: (C and D) Note an increased number of cells of all gastrointestinal layers, HE (Bars = 62 μm and 32 μm, respectively); (E) Similarly, we can note an increased number of cells of all gastrointestinal layers where a focal cellular exudates (white arrow) where it becomes contiguous into the three gastrointestinal layers (lamina propria, muscularis mucosae and submucosal), HE (Bar = 32 μm); (F and G) Higher magnification of the previous picture: In (F) note parasitized macrophages in the lamina propria (mucosa layer) (white arrows), (Bar = 16 μm) and in (G) observe macrophages loaded with *Leishmania *in the muscularis mucosae (white arrowheads) and submucosal layers (black arrowheads) (Bar = 16 μm); (H) Multinucleated giant cells (white arrows) or epithelioid cells (black arrowhead) formation can be seen associated to the exudate of mononuclear concentrated in lamina propria (plasma cells - small white arrow, lymphocytes - small black arrow), HE (Bar = 16 μm); LP: Lamina Propria; MM: Muscularis Mucosae; SM: Submucosal; GI: Glands of Intestine Mucosal) and Hematoxilin-Eosin (HE)**.

In infected animals, an increased number of cells, focal or diffuse, was observed in lamina propria, muscularis mucosae and submucosa of GIT, while no changes were found in the control dogs. A chronic cellular exudate was observed in all cases of Groups I and II and was composed predominately by macrophages, plasma cells and lymphocytes with rare polymorphonuclear neutrophils or eosinophils (Figure 1A-E). Macrophages containing many *Leishmania *amastigotes were easily found in lamina propria of the mucosa and the submucosa in the majority of the cases. Many of the macrophages showed a pale and abundant cytoplasm and less dense nucleus typical of epithelioid cells (Figure 1F and 1G). Multinucleated giant cells and epithelioid cells were associated with areas in which mononuclear cells were more concentrated, but no typical granulomatous reaction was evident in the lamina propria (Figure 1H).

**Figure 2:**
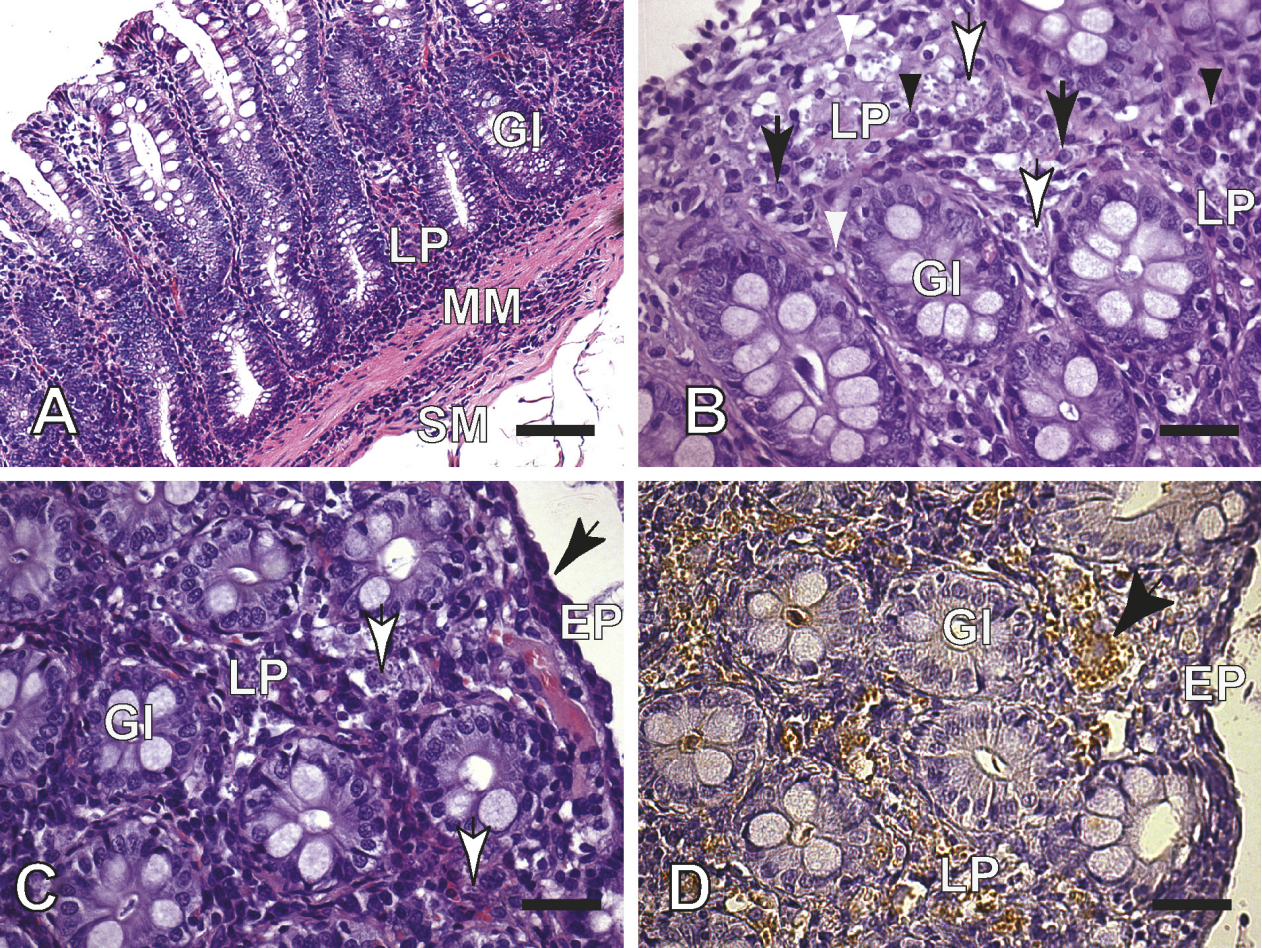
**(A-D) Colon sections of an asymptomatic dog naturally infected with *L. infantum***: (A and B) (A) Panoramic view of mucosal (lamina propria), muscularis mucosae and submucosal layers of caecum fragment showing an intense and diffuse cellular exudates, HE (Bar = 32 μm); (B) Higher magnification showing mononuclear cells represented by parasitized macrophages with *Leishmania *(white arrow) or not (black arrow), lymphocytes (white arrowheads) and plasma cells (black arrowheads) without ulcer or erosions of the epithelium (Bar = 16 μm); (C) Mucosa (Lamina propria) layer showing an increased number of cells. Macrophages with *Leishmania *can be noted (white arrows). Only a discrete atrophy (flatting) of the epithelial cells can be observed (black arrow), HE (Bar = 16 μm); (D) Same fragment of colon showing higher parasitism tissue load. Observe a hipertrophic macrophages with many intracellular forms of *Leishmania *(black arrow), Streptoavidin-peroxidase, (Bar = 16 μm). LP: Lamina Propria; MM: Muscularis mucosae; SM: Submucosal; GI: Glands of Intestine Mucosal and Hematoxilin-Eosin (HE).

Under microscopic analysis, we also observed that in spite of the parasitism we did not find mucosal erosions. Independently of the defined clinical status of the infected dogs, microscopic analysis revealed the presence of amastigote forms of *Leishmania *in all GIT layers, particularly in the cells (macrophages) of lamina propria (Figure 2A-D) without severe GIT mucosal alteration. In fact, immunolabeled amastigotes were evident in all intestinal segments, but the parasitism was more evidenced (frequency) in the caecum and colon than in other GIT segments. In addition a higher parasite load of amastigote forms of *Leishmania *were evident in these segments (Table 1). However, no statistical difference was observed between Groups I and II (Table 2). Thus, in clinically unaffected as well as the affected dogs, parasites were most numerous in the colon, followed by the caecum, while the other GIT segments contained relatively few parasites (Tables 3 and 4, respectively).

**Table 1:** Frequency of amastigote forms of *Leishmania *in the gastrointestinal tract (GIT) of asymptomatic and symptomatic naturally infected dogs with *Leishmania infantum *from Municipality of Ribeirão das Neves, Belo Horizonte Metropolitan area, Minas Gerais (MG) state, Brazil

Gastrintestinal fragments	Frequence (n = 20)	
	**Asymptomatic (n = 9)**	**Symptomatic (n = 11)**
	
Stomach	3 (33,3%)	1 (9,0%)
Duodenum	4 (44,4%)	6 (54,5%)
Jejune	5 (55,5%)	7 (63,6%)
Ileum	5 (55,5%)	7 (63,6%)
Cecum	8 (88,8%)	9 (81,8%)
Colon	6 (66,6%)	10 (90,9%)

**Table 2:** Morphometrical analysis (μm^2^) to quantify amastigotes forms of *Leishmania *in the gastrointestinal tract (GIT) of asymptomatic and symptomatic naturally infected dogs with *Leishmania infantum *from Municipality of Ribeirão das Neves, Belo Horizonte Metropolitan area, Minas Gerais (MG) state, Brazil

**Gastrintestinal fragments**	**Clinic status** **(n = 20)**		**Statistical analysis** **(Mann Whitney Test)**
	
	**Asymtomatic**	**Symptomatic**	**(p < 0,05)**
		
Stomach	0,21	0,15	0,9671
Duodenum	0,22	0,43	0,4029
Jejune	0,10	0,05	0,6474
Ileum	0,04	0,13	0,2443
Cecum	0,76	3,03	0,7725
Colon	0,41	4,73	0,1165

**Table 3:** Morphometrical analysis (μm^2^) to quantify amastigotes forms of *Leishmania *in the gastrointestinal tract of asymptomatic naturally infected dogs with *Leishmania infantum *from Municipality of Ribeirão das Neves, Belo Horizonte Metropolitan area, Minas Gerais (MG) state, Brazil

Gastrintestinal fragments	μm^2 ^	Gastrintestinal fragments	μm^2 ^	Statistical analysis (Friedman test) (p < 0,05)
Stomach	0,21	Duodenum	0,22	0,3383
Stomach	0,21	Jejune	0,11	0,4911
Stomach	0,21	Ileum	0,04	0,3678
Stomach	0,21	Cecum	0,76	0,2475
Stomach	0,21	Colon	0,41	0,9579
Duodenum	0,22	Jejune	0,10	0,5577
Duodenum	0,22	Ileum	0,04	0,7091
Duodenum	0,22	Cecum ^+ ^	0,76	0,0354*
Duodenum	0,22	Colon	0,41	0,2892
Jejune	0,10	Ileum	0,04	0,5589
Jejune	0,10	Cecum ^+ ^	0,76	0,0403*
Jejune	0,10	Colon	0,41	0,5607
Ileum	0,04	Cecum ^+ ^	0,76	0,0180*
Ileum	0,04	Colon	0,41	0,2667
Cecum	0,76	Colon	0,41	0,2928

**Table 4:** Morphometrical analysis (μm^2^) to quantify amastigote forms of *Leishmania *in the gastrointestinal tract of symptomatic naturally infected dogs with *Leishmania infantum *from Municipality of Ribeirão das Neves, Belo Horizonte Metropolitan area, Minas Gerais (MG) state, Brazil

Gastrintestinal fragments	μm^2 ^	Gastrintestinal fragments	μm^2 ^	Statistical analysis (Friedman test) (p < 0,05)
Stomach	0,15	Duodenum	0,43	0,4687
Stomach	0,15	Jejune	0,05	0,1387
Stomach	0,15	Ileum	0,05	0,6451
Stomach	0,15	Cecum	0,05	0,0761
Stomach	0,15	Colon ^+ ^	0,05	0,0302*
Duodenum	0,43	Jejune	0,05	0,7917
Duodenum	0,43	Ileum	0,13	0,6919
Duodenum	0,43	Cecum	3,03	0,0565
Duodenum	0,43	Colon ^+ ^	4,73	0,0138*
Jejune	0,05	Ileum	0,13	0,3912
Jejune	0,05	Cecum^+ ^	3,03	0,0125*
Jejune	0,05	Colon^+ ^	4,73	0,0031*
Ileum	0,13	Cecum^+ ^	3,03	0,0486*
Ileum	0,13	Colon^+ ^	4,73	0,0417*
Cecum	3,03	Colon	4,73	0,6457

## Discussion

Histology demonstrated an increased number of macrophages frequently parasitized with *Leishmania *amastigotes, plasma cells and lymphocytes throughout the GIT layers of all segments of the Groups I and II. However, parasites were predominantly located in the mucosa (lamina propria) of the GIT layers. These results are in accordance with Anderson et al. [28], Longstaffe and Guy [40] and Toplu et al. [36].

When comparing the GIT segments of all infected dogs we demonstrated the highest parasite load in the ceacum and colon. These results are consistent with Keenan et al. [41] González et al. [33] Ferrer et al. [30] and Adamama-Moraitou et al. [31]. Keenan et al. [41] observed comparable parasitological loading in all GIT segments of two distinct groups of German shepherd dogs experimentally infected with *L. chagasi *and *L. donovani*. González et al. [33] observed a severe chronic inflammatory process in the mucosa and submucosa of the colon and rectum of infected beagles where they described the surface and the epithelium of the crypts of Lieberkϋhn with progressive degeneration characterized by cellular swelling. As a result, focal micro-erosions developed in the mucosal surface reducing the area of the large bowel available for absorption and causing diarrhea. Therefore, the authors concluded that chronic colitis in beagles was caused by *L. infantum*, and that the diarrhea was consistent with a disorder prevailing in the large bowel. However, Ferrer et al. [30] reported chronic colitis and diarrhea in two dogs naturally infected with *L. infantum*, but questioned whether the concurrent lympho-plasmacytic infiltrate of the colonic mucosa was caused by *Leishmania *(detected by immunohistochemistry) or was an incidental finding, particularly relevant if the parasite load is low. The authors concluded that there was no evidence of a definitive pathogenic correlation between CVL and chronic colitis. Moreover, Adamama-Moraitou et al. [31] demonstrated that 32.3% of clinically affected dogs naturally infected with *L. infantum *presented with asymptomatic colitis. Endoscopic examination of colonic mucosa demonstrated focal hyperemia, edema and mild erosions in infected dogs, which were comparable to those observed in dogs with CVL or other infections including *Histoplasma, Salmonella *and *Yersinia*, or infected with parasites such as *Giardia, Trichuris, Ancylostoma, Entamoeba *and *Balantidium*. However, the authors excluded these infections after fecal examination. As reported by Chiapella [42], it is important to consider idiopathic large bowel inflammatory diseases such as plasmacytic-lymphocytic colitis and histiocytic-ulcerative colitis before making a diagnosis.

No severe macroscopic and microscopic lesions in the mucosa of the GIT during necropsy were evident during the present study. In some dogs the mucosa was somewhat hyperemic, and helminth parasites were observed in less than 20% of cases (three clinically affected and two unaffected dogs). Despite the presence of helminth parasites in the guts of some dogs, it was concluded that this did not interfere with the histological analysis of GIT segments from dogs with CVL, as no severe macroscopic or microscopic related alterations were evident, such as hemorrhages, ulcers or pyogenic granulomas. In the present study, an increased number of macrophages, frequently parasitized with *Leishmania *amastigotes, and plasma cells and lymphocytes, were observed throughout the GIT layers. However, as depicted in Figure 2, despite the presence of *Leishmania *amastigotes in macrophages of the lamina propria, only discrete atrophy of the mucosa epithelium cells without any severe lesions (necrotic or erosive mucosal lesions) was observed. González et al. [33] described the surface and the epithelium of the crypts of Lieberkϋhn as showing progressive degeneration characterized by cellular swelling, but with only focal micro-erosions, in the mucosa of the colon and rectum. Epithelioid and multinuclear cell formations were observed in some cases, although typical granulomas were not observed. However, Adamama-Moraitou et al. [31] reported that a granulomatous inflammatory pattern was the most common histological feature in the colonic mucosa and that this could be associated with a mildly eroded colonic mucosa.

In our study, clinically affected dogs harbored more parasites in the caecum and colon than in other GIT segments. This is consistent with other reports Anderson et al. [28]; Ferrer et al. [30]; González et al. [33]; Silva et al. [35]; Longstaffe and Guy [40]; Keenan et al. [41] where the difference in parasitic loading between specific GIT segments was observed in clinically affected dogs. However, it is important to reiterate that in our studies also clinically unaffected dogs harbored parasites in all GIT segments.

## Conclusion

We observed a high parasite burden throughout the GIT mucosa, but the pathological changes were relatively mild. Thus, this may led us to consider whether *Leishmania *gains an advantage from the intestinal immunoregulatory response (immunological tolerance). Such question will require further profound research and will help to elucidate the mechanisms underlying *Leishmania *infection.

## Competing interests

Research was supported by grants Conselho Nacional de Desenvolvimento da Pesquisa Tecnológica e Científica (CNPq/473601/2009-5) and Fundação de Amparo a Pesquisa do Estado de Minas Gerais (FAPEMIG/CBB 00219/09-80 and APQ-01355-09), Brazil. Students fellowship financed by Conselho Nacional de Desenvolvimento da Pesquisa Tecnológica e Científica (CNPq) and Coordenação de Aperfeiçoamento de Pessoal de Nível Superior (CAPES), Brazil. We do not have any kind of commercial interesting or competition with other researches. It is only an academic manuscript.

## Authors' contributions

AJWP, MMF and FLS did all the clinical exams, necropsies and histology. AJWP, MMF, TM did morphometrical and statistical analysis. MSMM was responsible for all serological exams. WLT, WLT advisors, revise all the histological analysis and the manuscript.

All authors read and approved the final manuscript.
